# Fecal Microbiota Transplantation: What’s New?

**DOI:** 10.3390/microorganisms10010023

**Published:** 2021-12-24

**Authors:** Luca Masucci, Gianluca Quaranta

**Affiliations:** 1 Dipartimento di Scienze di Laboratorio e Infettivologiche, Fondazione Policlinico Universitario A. Gemelli IRCCS, 00168 Rome, Italy; gianluca.quaranta@unicatt.it; 2 Dipartimento di Scienze Biotecnologiche di Base, Cliniche Intensivologiche e Perioperatorie, Università Cattolica del Sacro Cuore, 00168 Rome, Italy

The gut microbiota is composed of trillions of different microorganisms: bacteria, archaea, phages and protozoa, which represent a real solid organ, with an approximate weight of 2 kg. Specifically, our gastrointestinal tract harbors about 10^13^–10^14^ bacterial cells [1]. The most represented phyla are Firmicutes, Bacteroidetes and Proteobacteria, while Actinobacteria contribute less to the total bacterial composition [2]. Next-Generation Sequencing is highlighting new evidences on bacterial genes composition. In fact, bacteria encode 100-fold more genes than the human genome. This complex of bacterial genes is defined as microbiome. These genes directly impact on antibiotic resistance, drug side effects, biofilm formation, immunity modulation, nutrition and health [3]. The equilibrium between bacterial communities and their gene regulation plays a key role in the maintenance of nutritional and immunological functions. One of the main properties of gut microbiota is the ability to respond, within physiological limits, to damaging events. With good reason, many efforts have focused on “exploiting” this bacterial elasticity with the aim to intervene on the composition of the intestinal microbiota and, therefore, on general health status. Fecal microbiota transplantation (FMT) represents one of the most innovative therapeutic approaches for gut microbiota modulation. FMT consists in the infusion of fecal suspension from a healthy donor into a recipient patient in order to restore the microbial population equilibrium also known as “eubiosis” [4]. The origins of this method go back to the 4th century, when an ancient Chinese medicine practitioner, named Ge Hong, used a fecal suspension called “yellow soup” to treat his patients with severe diarrhea via oral administration [5]. The history of FMT has continued down to the 17th century, when veterinarians used stool as a therapeutic option for livestock farms. During World War II, camel stool was also used by German soldiers to treat bacterial dysentery. The modern era of FMT as a therapy can be traced back to 1958, when Eiseman and colleagues treated four cases of pseudomembranous colitis by using a fecal suspension administered via enema [6]. In the last decade, FMT bounced back dramatically as a valid therapeutic solution, particularly to treat *Clostridioides difficile* infection (CDI). In fact, the intestinal microbiota of CDI patients is characterized by a marked increase in Proteobacteria and a strong decrease in the Firmicutes/Bacteroidetes ratio. Given this, the primary goal of fecal transplantation is reversing this microbial pattern, re-establishing a state of eubiosis. Besides intestinal diseases, reports have shown benefits of FMT also in several systemic disorders. Considering the described gut–brain axis and the complex pathways exerted by microbial population, FMT has become a charming tool for the treatment of extra-intestinal disorders, such as insulin resistance, metabolic syndrome, irritable bowel syndrome, atherosclerosis, obesity, multiple sclerosis, Parkinson’s disease and hepatic encephalopathy. By now, several species have been elected as “good” bacteria. Interesting examples are *Akkermansia muciniphila, Faecalibacterium prausnitzii, Bifidobacterium* spp., *Lactobacillus* spp., and *Eubacterium* spp., which relative abundance is linked to a healthy gut. On the other side, “bad” bacteria such as *Fusobacteriun nucleatum, Bacteroides fragilis* toxin-producing, *Prevotella copri* and many others are evaluated as a potential contributing cause of systemic disorders. Future trials are needed to clarify this simplistic “good” or “bad” labeling and to understand how FMT impacts microbial population and subsequent clinical outcome improvement. 

The FMT procedure presents several critical points to overcome. First, the recruitment of a healthy donor often appears very difficult. In fact, the donor is first submitted to a medical questionnaire and then to serological and microbiological screening tests. If all requirements are fulfilled, the donor will be defined as “suitable”. The donated fecal material will be further examined to avoid any possible transmission of pathogens to the recipient. Another important critical point concerns the organizational and bureaucratic management, differently encoded in each country. Moreover, the whole procedure involves several professional figures including microbiologists, laboratory technicians, gastroenterologists and nurses and their respective operating units. Orchestrating all human resources to a smooth workflow can be very complex.

A future goal must be focused on finding alternative forms of intestinal microbiota modulation that may be less complex in management and more comfortable for receiving patients. Concrete examples of this trend are the preparation of capsules containing fecal material which can be taken independently by the patient. A further alternative is represented by the bacterial consortium. Bacterial consortium is a suspension of bacteria, specially selected and cultured from a healthy donor. In this case, the administration could be performed via colonoscopy or oral tablets, bypassing the complexity of recruitment.

In the “personalized medicine era”, understanding the potential impact of the bacteria–host relationship is one of the principal aims for researchers. Once this correlation has been identified, the main challenge will be intervening on the intestinal microbiota in order to target its composition and, consequently, the functional outputs. Recently, among the many studies, several are focused on the microbiota–gut–brain axis (MGBA). For example, Autism spectrum disorders (ASD) could be associated to this route. A recent study used FMT to evaluate the efficacy of the infusion in forty ASD patients, showing an improvement of related symptoms [7].

FMT procedure derives from ancient alchemy and represents a mostly empirical approach. A special issue gathering all the current knowledge and expertise about clinical and laboratory facilities could clarify and spread the encouraging potentialities of this therapeutical option. 
